# Evaluation of Additives on the Cell Metabolic Activity of New PHB/PLA-Based Formulations by Means of Material Extrusion 3D Printing for Scaffold Applications

**DOI:** 10.3390/polym16192784

**Published:** 2024-09-30

**Authors:** Ivan Dominguez-Candela, Lluc Sempere-José, Ignacio Sandoval-Perez, Asunción Martínez-García

**Affiliations:** AIJU Technological Institute for Children’s Products & Leisure, 03440 Ibi, Spain; llucsempere@aiju.es (L.S.-J.); nachosandoval@aiju.es (I.S.-P.); sunymartinez@aiju.es (A.M.-G.)

**Keywords:** additive manufacturing, scaffold, polyhydroxyalkanoates, material extrusion, bio-based additive, bone regeneration, metabolic activity, mechanical properties

## Abstract

In this study, specific additives were incorporated in polyhydroxyalcanoate (PHB) and polylactic acid (PLA) blend to improve its compatibility, and so enhance the cell metabolic activity of scaffolds for tissue engineering. The formulations were manufactured through material extrusion (MEX) additive manufacturing (AM) technology. As additives, petroleum-based poly(ethylene) with glicidyl metacrylate (EGM) and methyl acrylate-co-glycidyl methacrylate (EMAG); poly(styrene-co-maleic anhydride) copolymer (Xibond); and bio-based epoxidized linseed oil (ELO) were used. On one hand, standard geometries manufactured were assessed to evaluate the compatibilizing effect. The additives improved the compatibility of PHB/PLA blend, highlighting the effect of EMAG and ELO in ductile properties. The processability was also enhanced for the decrease in melt temperature as well as the improvement of thermal stability. On the other hand, manufactured scaffolds were evaluated for the purpose of bone regeneration. The mean pore size and porosity exhibited values between 675 and 718 μm and 50 and 53%, respectively. According to the results, the compression stress was higher (11–13 MPa) than the required for trabecular bones (5–10 MPa). The best results in cell metabolic activity were obtained by incorporating ELO and Xibond due to the decrease in water contact angle, showing a stable cell attachment after 7 days of culture as observed in SEM.

## 1. Introduction

Nowadays, additive manufacturing (AM) technologies are considered one of the most crucial manufacturing methodologies. The freedom in terms of design for obtaining complex structures, easy and fast designing, low-cost as well as rapid prototyping, are examples of the main advantages of this technology [1]. Among the available AM techniques, the material extrusion (MEX) is, by far, the largest employed, which can also be found or known as 3D printing in general [2]. Over the recent years, this has been applied to different sectors, such as the automotive, construction or biomedical sectors. The latter has gained more interest due to the worldwide issue that the social population is facing. The increase in bone fractures because of diseases or accidental injuries, particularly for the older population, has increased the need to regenerate and repair the bone tissue [3]. For instance, bone fractures occurred every 20 seconds as a consequence of osteoporosis in people over 50 years [4].

In order to overcome this social issue, MEX has emerged to present the capability to design and manufacture customized scaffold for tissue engineering or prostheses, among others [5]. Even though petroleum-based polymers, such as polyethylene (PE), have been widely used in implant applications because they are biologically inert polymers [6], the current trend focuses on biocompatible and biodegradable/bioresorbable polymers once the bone has grown. The bioresorbability of polymers gives them the ability to degrade into by-products that are non-toxic and are eliminated by natural pathways [7]. These types of polymers are targeted to fabricate temporary implants, which avoids the need for a second intervention for its removal [8]. Among the polymers for tissue engineering, the most used are polycaprolactone (PCL) and poly(lactic acid) (PLA). On one hand, PCL is a synthetic biomaterial that is usually applied to long-term applications since its hydrophobic behaviour make it more stable for reabsorption by the human organism [9]. On the other hand, PLA, which is a biopolymer produced by fermentation from renewable resources such as sugar cane or corn, is more easily reabsorbable for its better hydrophilicity compared to PCL [10]. The use of PLA is approved by the US Food and Drug Administration (FDA) for clinical and biomedical applications [11]. Nevertheless, PLA present some disadvantages that must be addressed in order to manufacture efficient scaffolds; for example, the insufficient cellular adhesion on its surface and low osteoconductivity [12]. In addition, despite being a biocompatible polymer, the PLA degradation process can generate an inflammatory response and toxicity because of the release of acidic species [13]. As an alternative polymer for tissue engineering, the polyhydroxyalkanoates (PHAs), in particular polyhydroxybutyrate (PHB), are gaining more interest for their renewable origin, biodegradability and compatibility with the human body, leading to support cell growth in tissue formation. In fact, PHA degradation has shown a less inflammatory response than PLA [14] as well as a degradation rate that is suitable for the development of scaffolds. However, PHB exhibited an inherent high degree of crystallinity that make it brittle as well as having a poor narrow processing window and processability.

The blending process is a promising proposal to enhance PHB properties. In this sense, the incorporation of PLA has been studied to improve processibility during the 3D printing process and thermal stability [15]. Regarding the degradation in vitro for tissue engineering, it has also been reported that the PHB/PLA blend leads to a more uniform enzymatic degradation compared to the pristine polymers individually, which is of great benefit [16]. Then, despite that PHB/PLA blend present good compatibility and bioreabsorbable capacity required to be used as scaffolds, there is still a need to improve its brittleness [17]. The incorporation of additives that act as compatibilizers or plasticizers is a proposal that has been carried out for researchers to improve the PLA/PHB blend. On one hand, the use of reactive compatibilizers is a current strategy to enhance the properties of blends. Mokrane et al. [18] reported the improvement of compatibilization in PLA/PHBHHx (75/25 wt%) blend reinforced with halloysite nanotubes (HNTs) and the addition of maleic anhydride as a compatibilizer. In other studies, dicumyl peroxide was incorporated in a PLA/PHB blend (75/25 wt%) during its processing in order to improve the miscibility of the blend, resulting in better thermal stability as well as enhancement of its mechanical properties [19]. On the other hand, the use of modified vegetable oils has gained more interest over the recent years due to their renewable origin and the presence of reactive groups within the chemical structure. It has been reported that its incorporation can provide both a plasticizer and compatibilizer effect, resulting in the improvement of miscibility and ductile properties [20]. For instance, Lopera-Valle et al. [21] employed epoxidized canola oil (ECO) at 5 and 10 wt% in a PHB/PLA blend (3:1), which showed that ductile properties increased as the ECO content was higher. However, to the best of our knowledge, there is not much literature about the effect of additives in the blend of PHB-rich phase (75 wt%) and PLA (25 wt%) and less for printing scaffolds by means of additive manufacturing in the tissue engineering sector.

Therefore, the aim of this study is to develop new biocomposites to be applied in 3D printing and to evaluate the effect of specific additives that have not been used before in a blend of PHB-rich phase with PLA in order to improve their compatibility for developing scaffolds. Concerning the additives, both petrochemical and bio-based compatibilizers have been employed for this purpose. Poly(ethylene-co-glycidyl methacrylate) (EGM), poly(ethylene-co-methyl acrylate-co-glycidyl methacrylate) (EMAG) and a random copolymer of styrene and maleic anhydride (Xibond) were selected to improve the interactions of PHB/PLA due to its functionalities. These are compared with a bio-based epoxidized linseed oil (ELO) that contains epoxy groups. First, the effect of compatibilization and/or plasticization in the blend has been evaluated by means of mechanical, thermal and morphological tests. Afterwards, morphological, mechanical, water angle contact and biological characterization of the designed porous scaffold were carried out.

## 2. Materials and Methods

### 2.1. Materials

Bacterial poly(3-hydroxybutyrate) (PHB) in pellet form was a commercial-grade ENMAT Y3000, obtained from TianAn Biopolymers (Beilun, China). The PHB has a density of 1.25 g·cm^−3^ with a DSC melting point at 175–180 °C. The aliphatic polyester poly(lactic acid) (PLA), commercial-grade Ingeo 3D 870 was supplied in pellet form by NatureWorks (Minneapolis, MI, USA). Its density is 1.22 g·cm^−3^ and its DSC melting point of 165–180 °C makes it suitable to be blended with the reported PHB.

**Figure 1:**
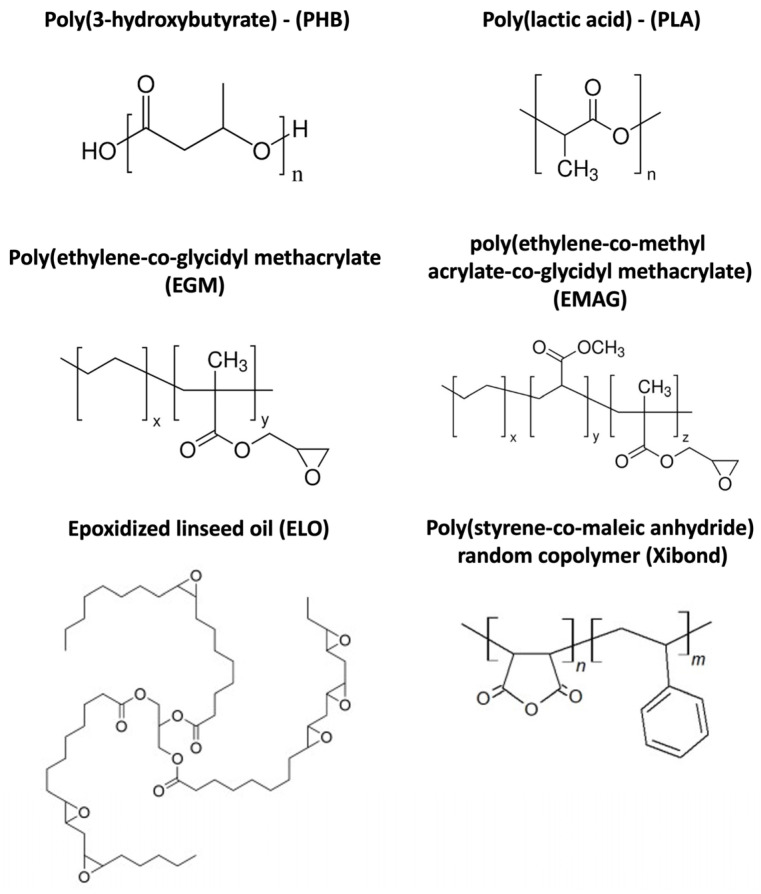
Chemical structure of matrices and compatibilizers employed.

Regarding compatibilizers, three petrochemical derivatives were used. Poly(ethylene-co-glycidyl methacrylate) (EGM) was acquired from Sigma Aldrich (Madrid, Spain). The EGM density is 0.94 g·cm^−3^ and its glycidyl methacrylate content is 8 wt%. The poly(ethylene-co-methyl acrylate-co-glycidyl methacrylate) (EMAG) was obtained from Sigma Aldrich (Madrid, Spain). Its density is 0.94 g·cm^−3^ with a glycidyl methacrylate content and methyl acrylate of 8 and 25 wt%, respectively. The additive Xibond 250 is a random copolymer of styrene and maleic anhydride kindly supplied by Polyscope (Geleem, The Netherlands). It presents a molecular weight (MW) of 10.000 g·mol^−1^, an acid value of 285 mg KOH·g^−1^ and a glass transition temperature of 130 °C. As a bio-based compatibilizer, epoxidized linseed oil (ELO) was produced by Traquisa SL (Barcelona, Spain). ELO presents an epoxy content of >8 wt%, density of 1037 g·cm^−3^ and MW of 1038 g·mol^−1^. The chemical structure of each polymer matrix and compatibilizer is shown in Figure 1.

### 2.2. Development of PHB/PLA Biocomposites

Different formulations were developed by extrusion compounding by using a twin-screw co-rotating extruder Collin Teach Line ZK25 from Collin Lab & Pilot Solutions GmbH (Maitenbeth, Germany). Before processing, both polymer matrices, PHB and PLA, were dried at 60 °C for 8 h as recommended by suppliers using an air-circulating oven Model 50-400 from DRI-AIR Industries, INC (East Windsor, NY, USA). The selection and amount of the incorporated additives were chosen based on the literature, which suggested that 5 phr showed a significant improvement of compatibilizer’s effect and mechanical properties [22,23,24,25]. Regarding the reactive groups found in selected additives, generally, the epoxy groups are one of the main groups considered for the purpose of compatibilization or plasticization because they can react easily with the hydroxyl and carboxylic groups of the PHB [26] and PLA [27] matrices. It should be pointed out that this is not a unique group used for this target, since others, such as maleated, which contains a maleic anhydride (found in Xibond additive), or an amine group that can be found in chitosan [28], are also able to react with PHB and PLA matrices. For this reason, the aforementioned additives were chosen for both the reasons of their specific reactive group and effectiveness, after considering the previously described literature reported by other authors. Then, the biocomposites were prepared according to the compositions included in Table 1, optimizing the parameters until setting a temperature profile of 165/180/185/185/180 °C from the hopper to the die at a constant screw rate of 65 rpm. Afterwards, each composition was pelletized with an air-knife unit Collin Teach Line SP cutter from COLLIN Lab & Pilot Solutions GmbH (Maitenbeth, Germany) and air-cooled at room temperature. Finally, the pellets were dried again at 60 °C for 8 h and stored in hermetic bags to prevent moisture uptake.

### 2.3. 3D Printing of PHB/PLA Biocomposites

**Figure 2:**
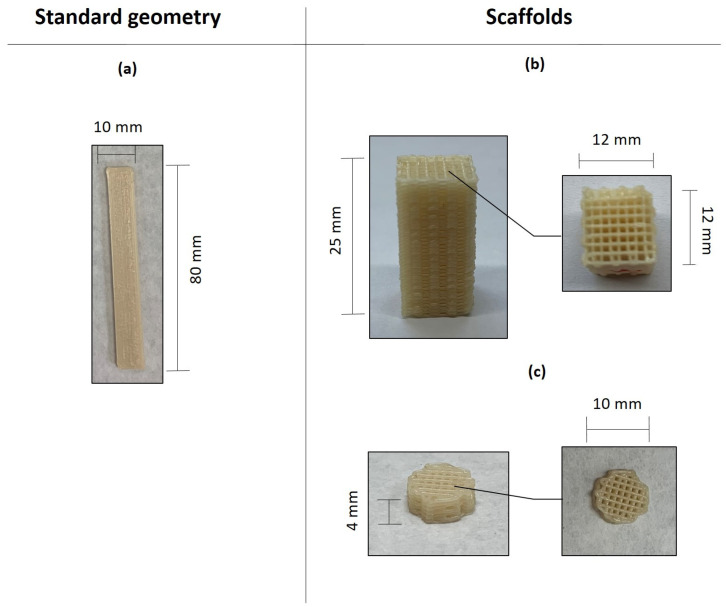
Standard geometries and scaffolds printed in this work: (**a**) flexural and impact test speciments (**b**) compression test specimens and (**c**) cell proliferation test.

The 3D printing was performed on a TUMAKER NX Pro Pellets (Utrecht, The Netherlands) based on the MEX process and using a nozzle of 0.4 mm. In this work, several geometries have been developed in order to assess the behaviour of the composites. On one hand, standard geometries were considered for the flexural and impact-absorbed energy test specimens with a size of 80 × 10 × 4 mm^3^, respectively. On the other hand, the scaffold specimens were fabricated with two different geometries to evaluate their compression behaviour (12 × 12 × 25 mm^3^) and cell proliferation test (diameter of 10 mm and thickness of 4 mm). The printing parameters are shown in Table 2 and the manufactured geometries in Figure 2. In all cases, seven replicates were printed for each formulation and test to obtain the average and deviation values.

The printed parts were designed using a computer-aided design software Autodesk Fusion 360, version 2.0.10806 (San Rafael, CA, USA). To set the parameters for the 3D printing, the software Simplify 3D version 4.1 (Cincinnati, OH, USA) was employed to generate the G-code file. The printed flexural and impact parts obtained by means of additive manufacturing were used to characterize the mechanical properties of the formulations developed. For this reason, the infill density and raster angle were set as 100% and 0/90°, respectively. In the case of scaffold manufacturing, two geometries based on a cube (Figure 2b) and a cylinder (Figure 2c) were selected. The printed parts were manufactured with an infill density of 30% in order to obtain a specific porosity (50% theoretical), which is within the range for bone regeneration [29,30]. The G-code file used for printing was set with no walls and no top/bottom layers and a raster angle of 0/90°.

### 2.4. Mechanical Characterization

The flexural tests of standard geometries were carried out following the guidance of ISO 178 [31]. For this purpose, a universal testing machine Instron 6025 from Instron (Barcelona, Spain) was employed, equipped with a 10 kN load cell and using a crosshead rate of 5 mm·min^−1^. The Charpy impact strength was measured in a Resil 5.5 impact testing device from CEAST RESILIMPACTOR (Cerdanyola Del Vallès, Barcelona, Spain) with a 1 J pendulum. The morphologies of the Charpy fractured samples were assessed by means of field scanning electron microscopy (FESEM). A JEOL SEM J840 (Akishima, Tokyo, Japan) was used with an acceleration voltage of 5 kV. Prior to observation, samples were coated with a thin layer (Pd-Au alloy) in a sputter coater EM MED020 from Leica Microsystems (Wetzlar, Germany) over 120 s.

For the scaffold characterization, the compression test was performed according to ISO 604 [32] using an Instron 6025 (Canton, MA, USA) with a crosshead rate of 5 mm·min^−1^ and a load cell of 10 kN. In this sense, the values of stress and deformation were taken from the yield point, which is where the curve starts to decrease [33].

### 2.5. Thermal Properties

The thermal behaviour of PHB75-PLA samples with additives were analyzed by differential scanning calorimetry (DSC) and thermogravimetric analysis (TGA). The thermal transitions of blends were evaluated using a DSC Q200 equipment from TA Instruments (New Castle, DE, USA). Samples with an average weight between 6 and 10 mg were evaluated with the following dynamic temperature program: a heating program from 25 to 200 °C followed by a cooling cycle from 200 to −40 °C was carried out in order to remove the thermal history. Finally, a heating program up to 200 °C was carried out at a rate of 10 °C·min^−1^. The thermal programs were performed using a nitrogen atmosphere with a flow rate of 66 mL·min^−1^. The degree of crystallinity was calculated using Equation (1) to provide a more accurate value of the considered blend (PHB/PLA) [34].

(1) $$X_{c}=\frac{\Delta H_{m}-\Delta H_{cc}}{\left(\Delta H_{mPHB\left(100\%\right)}\times w_{PHB}-\Delta H_{mPLA\left(100\%\right)}\times w_{PLA}\right)}\times 100$$

where $\Delta H_{m}$ is melt enthalpy of blend, $\Delta H_{cc}$ is cold crystallization enthalpy and both $w_{PHB}$ and $w_{PLA}$ are the weight proportion of PHB and PLA, respectively. The $\Delta H_{mPHB\left(100\%\right)}$ and $\Delta H_{mPLA\left(100\%\right)}$ corresponds to the melt enthalpy of a theoretical fully crystalline structure of PHB and PLA, whose values are 146 J·g^−1^ [35] and 93 J·g^−1^ [36], respectively.

The thermal stability evaluation was assessed by TGA analysis in a TGA Q500 equipment from TA Instruments (New Castle, DE, USA). The samples were subjected to a heating ramp from 30 to 700 °C at 10 °C·min^−1^. The nitrogen flow rate was set at 66 mL·min^−1^ and the average weight of samples was 10 mg. In order to assess the maximum degradation rate of the samples, derivative thermogravimetric curves (DTG) were first obtained and analyzed.

### 2.6. Water Contact Angle (WCA)

With the aim to evaluate the hydrophilic behaviour, flat-surface samples were manufactured to measure the WCA using the sessile drop method, as recommended [37]. Samples of 60 × 60 × 1 mm^3^ were obtained by means of compression moulding using a Hydraulic Press 20 from Lab Tech Engineering Company LTD (Samutprakarn, Thailand). The process set-up was as follows: heat up to 195 °C and apply pressure of 10 bar maintained over 5 min; subsequently, the pressure is released, and the sample air-cooled at room temperature. The WCA measurement was carried out through a content angle goniometer model 100-00 from Ramé-hart Instrument Co (Succasunna, NJ, USA). To assess the static contact angle, a 2 μL of distilled water was dropped on the non-porous surface of samples and analyzed after 2 s using a software Drop Image version 2.10.00. An average measurement of 6 contact angles was performed per composition.

### 2.7. Morphological Characterization

The scaffold porosity and surface morphology with dimensions of 10 mm diameter and 4 mm of thickness was evaluated. The estimated porosity was obtained by gravimetric method which is the most commonly used in the literature [37]. The total porosity was calculated with the Equation (2):

(2) $$\mathrm{Total}\;\mathrm{porosity}=\left(1-\frac{\rho_{ap}}{\rho_{bulk}}\right)\times 100$$

where $\rho_{ap}$ is the density of the scaffold obtained by the apparent volume, and $\rho_{bulk}$ is the density of bulk material measured in a density determination kit for xs140 balance with a resolution of 0.01 g cm^−3^ from Mettle Toledo (Barcelona, Spain).

The pore size was evaluated by considering the distance between the filaments of the printing pattern (0/90°). The measurements were obtained using a Hitachi TM3030 SEM (Hitachi, Tokyo, Japan) with an acceleration voltage of 5 kV.

### 2.8. Cell Seeding and Culture

To evaluate the cell behaviour in the formulations developed, a human osteoblastic cell line hFOB 1.19 (ATCC ^®^ CRL-11372TM) was employed. Four samples of 3D porous scaffold of each formulation were manufactured for this purpose with dimensions of 4 mm in height and 10 mm in diameter. The employed cell-culture medium consisted of a mixture 1:1 of DMEM and Ham’s F-12 without fenol red (Dulbecco’s Modified Eagle Medium/Nutrient Mixture F-12 with high glucose, L-glutamine, Gibco). It was supplemented with 10% of Foetal Bovine Serum (FBS) and 0.3 mg·ml^−1^ of antibiotic–antimycotic solution (Gibco, Life Technologies, Carlsbad, CA, USA). The dimensions of the flask used for cell culture were 75 cm^2^ (T75) from Sarstedt (La Roca del Vallès, Spain) until values of 80–90% confluence were achieved. Once these values were reached, cells were removed with trypsin-EDTA 0.5% (no phenol red) from HyClone (Washington D.C, Columbia, USA).

The 3D printed porous scaffolds of each formulation were quadruplicated, sterilized by using ethanol 75% *v*/*v* followed by UV light exposure under fume hood during 30 min. Consequently, samples were washed three times with PBS (59321C Dulbecco’s Phosphate Buffered Saline) from Merck (Darmstadt, Germany) and with culture medium in order to remove possible traces of ethanol. Afterwards, the scaffolds were placed individually in sterilized centrifuge tubes (CFT011150, 15 mL sterile tubes) from Jet Biofil (Guangzhou, China) and were seeded with 50 µL cell suspension containing a density of around 93,000 cells. Then, scaffolds were kept for 1 h in the CO_2_ incubator with 5% of CO_2_ at 37 °C and then 2 mL of culture medium were added to the tubes to provide enough nutrient for the first 24 h of cell culture.

### 2.9. Cell Metabolic Activity Evaluation

With the aim to evaluate the cell metabolic activity, a CCK-8 protocol (Cell Counting kit-8) from Dojindo Molecular Technologies (Kanagawa, Japan) was used. For the 1st day of culture, samples of each formulation were transferred from tubes to a non-treated 24-well plate from Thermo Scientific ^TM^ Nunc TM (Waltham, MA, USA) that were kept until the end of experiment. Each scaffold was washed using 1 mL of PBS and supplemented by a 1 mL of CCK-8 solution at 10% *v*/*v*. A similar procedure was carried out with 4 empty wells, use it as negative controls. The plate was incubated at the following conditions: 37 °C, 5% CO_2_ for 4 h. After the incubation, two aliquots of 100 µL were transferred from each well to a 96-well reader plate from Thermo Scientific ^TM^ Nunc-Immuno ^TM^ Microwell ^TM^ (Waltham, MA, USA). In order to measure the absorbance, a BioTek ELx800 reader (Bio Tek Instruments Inc., Winooski, VY, USA) was used to read the excitation wavelength of 450 nm. Once the absorbance had been measured, the solution that contained CCK-8 was removed from the culture plate, washed three times with PBS and 2 mL of supplement media was added before again incorporating the plate in the incubator. The same procedure was repeated at 4 and 7 days of cell culture, replacing the culture medium every day.

After 7 days of culture, the cell-seeded scaffolds were subjected to wash with PBS solution (Sigma Aldrich, St. Louis, MO, USA) and fixed with 2.5% glutaraldehyde (Sigma Aldrich, St. Louis, MO, USA) solution in PBS at 4 °C for 1 h. Then, samples were dehydrated using ethanol solutions of 30, 50, 70, 90 and 100% *v*/*v* and treated with hexamethyldisilane. In order to evaluate the cell morphology, scaffolds were cut longitudinally to observe the interior. Prior to observation, the samples were coated with Pd/Au for 120 s with 18 mA under vacuum using a Polaron SC7620 (quorumtech, sputter Coater, East Sussex, UK). The cell morphology was observed in a FESEM Zeiss Sigma 300VP (Oxford Instruments, Oxforshire, UK) with an acceleration voltage of 1 kV.

## 3. Results

### 3.1. Effect of Additives in PHB/PLA Blend

As indicated in Section 2.3, with the aim to assess the effect of the additives in a PHB/PLA blend (PHB75-PLA), a standard geometry, shown in Figure 2a was manufactured with the infill structure and printing parameters included in Table 2.

#### 3.1.1. Mechanical Properties of the PHB/PLA Blends

In order to evaluate the mechanical properties, flexural tests of 3D printed samples have been manufactured following the recommendation of R Donate et al. [38], which evaluate the mechanical properties of formulation targeted for scaffold manufacturing by means of flexural tests of non-porous samples. The results are shown in Table 3.

In the case of flexural modulus, the highest values were obtained by the pristine PHB75-PLA sample with a value of 3330 MPa. Nevertheless, incorporating additives such as EGM, EGMA, and ELO leads to a slight decrease in this property for values up to between 2900 and 3080 MPa, regardless of the additive employed. The latter’s effect is observed due to the improvement of ductility that will be explained later, thus triggering a decrease in rigidity and, therefore, a flexural modulus as expected. In the case of Xibond additive, a similar flexural modulus as the PHB75-PLA sample was observed without significant differences. Regarding flexural strength, the values remain unchanged except for Xibond, which provided the highest value (57.0 MPa) compared to 53.5 MPa of the pristine PHB75-PLA sample. This behaviour can be attributed to the compatibilizing effect that Xibond provides, whose maleated groups interact with the hydroxyl and carboxylic groups of PHB and PLA, suggesting a stronger chemical interaction between Xibond and a polymer blend matrix that enables the flexural strength to increase. A similar finding has been reported by Tejada-Oliveros et al. [39] when compatibilized the PLA/polycarbonate (PC) blend with the same copolymer Xibond manufactured by injection moulding. Regarding flexural strain, a value of 3.1% was observed in the pristine PHB75-PLA. Both EGM and EMAG exhibited a slight enhancement of flexural strain. On the one hand, the slight improvement in flexibility of PHB75-PLA-EGM can be explained by the interaction between the glycidyl methacrylate group within the EGM structure and the hydroxyl and carboxylic groups in PHB and PLA, respectively. On the other hand, the higher flexural strain observed in the PHB75-PLA-EMAG sample compared to the EGM additive can be due to the presence of two reactive groups that contain EMAG additive (acrylate and glycidyl methacrylate group), enhancing the interaction with the PHB/PLA blend. According to Chang et al. [40], the increment of the ductile properties also indicates an improvement of compatibilization between blends as a consequence of chain interactions. These results agree with the decrease in tensile modulus and strength obtained using these two additives in comparison with the pristine sample. In the case of the ELO additive, it reached the highest flexural strain value with an improvement of around 32% compared to the PHB75-PLA sample. This behaviour is somehow expected, since modified vegetable oils can provide a dual compatibilizing and plasticizing effect in the blend. In this study, the epoxy group that contains the ELO structure can link with the hydroxyl and carboxilic groups available in the aliphatic polyester chains (PHB and PLA), which can favour both a plasticizing and compatiblizing effect in the blend [41]. This dual effect is observed not only for the slight improvement in the average value on flexural strength but also for the increment of the ductility. In this sense, Yi Han et al. [42] also reported both effects when employing 5 phr of epoxidized soybean oil (ESO) in the PLA/PBAT blend, showing an increase in yield strength followed by improvement of elongation at break. Finally, the Xibond additive slightly improved the mechanical resistance of the sample, characterized by the highest flexural strength compared to the PHB75-PLA sample, as aforementioned. As a result, the flexural strain did not improve significantly with incorporating the additive, indicating that Xibond leads to an improvement in rigidity more than ductility, likely due to the presence of a maleated group in the structure.

**Figure 3:**
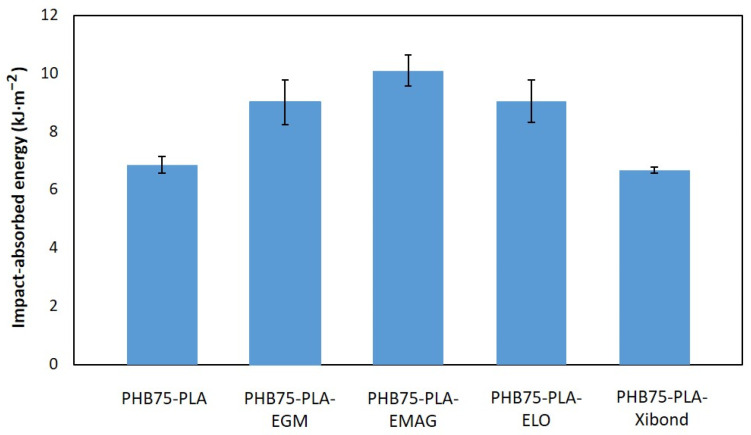
Impact-absorbed energy from the Charpy impact test of PHB75-PLA blend samples with additives.

The impact-absorbed energy of PHB75-PLA samples with additives employing standard geometries is shown in Figure 3. As initially expected, the pristine PHB-rich sample (PHB75-PLA) exhibited the lowest impact absorption energy with a value of 6.85 kJ·m^−2^, being close to the pristine PHB (5.4 kJ·m^−2^) reported in the literature [43]. By incorporating both polyethene derivates, EMAG seemed to provide better-absorbed energy than EGM with improvements of 47.2 and 31.4%, respectively, compared to the PHB75-PLA sample. This result is in concordance with the flexural test obtained, where EMAG show a higher elongation at the break and flexural modulus. This effect could be ascribed to a better compatibilizing effect as a consequence of both the reactive groups acrylate and glycidyl methacrylate in the structure, providing an enhancement of absorbed energy in a high strain rate test. The bio-based ELO achieved an improvement in impact-absorbed capacity from 6.85 for pristine PHB75-PLA to 9.00 kJ·m^−2^ for PHB75-PLA-ELO because of its dual effect as plasticizer and compatibilizer, as aforementioned in the flexure test. In this regard, no significant differences were found with respect to EGM and EMAG after considering its standard deviations. For the PHB75-PLA-Xibond sample, a significant increase in impact-absorbed energy was not observed under the Charpy test despite its improvement in mechanical properties from the flexural test. A similar behaviour was exhibited in the PA1010/PLA blend (80:20 wt%) when 2 phr of poly(styrene-ran-glicidyl methacrylate), with a similar chemical structure to Xibond but with a different reactive group, was incorporated in order to improve the compatibility of the blend. In this case, the impact strength remained constant but, in contrast, an increment of 8.16 and 18% in flexural modulus and stress was recorded, respectively [44].

**Figure 4:**
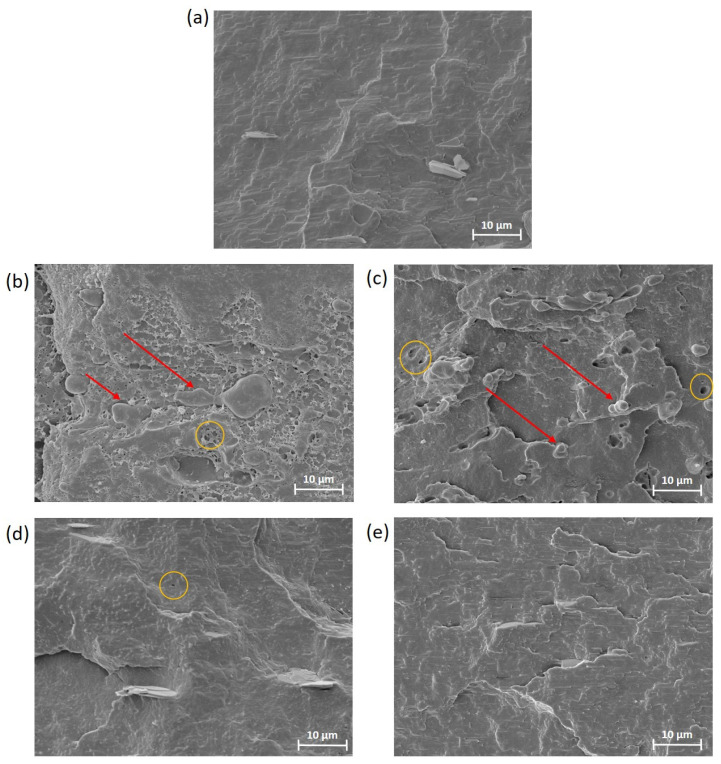
SEM images of impact fractured samples by field emission electron microscopy (FESEM) of PHB75-PLA blend samples with additives: (**a**) PHB75-PLA, (**b**) PHB75-PLA-EGM, (**c**) PHB75-PLA-EMAG, (**d**) PHB75-PLA-ELO and (**e**) PHB75-PLA-Xibond. The dispersed phase and cavities are represented by the red arrows and yellow circles, respectively.

The fracture morphology of PHB75-PLA samples with additives obtained after the Charpy impact test was evaluated by SEM at 2000×, as shown in Figure 4. For PHB-rich phase formulation (PHB75-PLA), it can be seen that the surface showed a rough fracture characteristic of the PHB matrix but without the presence of the well-known wavy surface of the pristine PHB, since it consists of a blend of PLA with PHB-rich phase that results in this morphology [45]. In this case, the presence of voids was not found, which indicates a good dispersion despite it not having been reported that PLA/PHB formulation presents an immiscible and/or partially miscible nature [46]. This fact could indicate that the processing and manufacturing conditions in material extrusion 3D printing were good enough to ensure a correct mixing of the blend. From Figure 4b, it can be seen that incorporating EGM showed a presence of dispersed phase, highlighted by red arrows, as was expected. Nevertheless, the low presence of small-sized cavities after the impact test in the fractured surface, highlighted by yellow circle, indicates a good interfacial adhesion between both matrixes (PHB and PLA). The surface exhibited a rougher fracture as well as the presence of filaments, which could suggest an interaction of matrixes due to its compatibilization, as has been reported in other studies. For instance, a similar morphology was found in the compatibilization study of PLA with an olefin block copolymer using a random terpolymer of ethylene–methyl acrylate–glycidyl methacrylate (GMA) as compatibilizer [47]. In addition, this morphology is in agreement with the mechanical properties that exhibit an increment of absorbed energy after the impact test compared to the PHB75-PLA sample. The incorporation of EMAG showed a lower presence of dispersed phase with a smaller diameter as well as a better homogeneity in the matrix than GMA incorporation, which can suggest a better compatibility between PHB and PLA. In this regard, it correlates with better mechanical properties than the material with EGM. In the case of modified vegetable oil, ELO, a rougher surface was observed with respect to the sample without additives. This surface is characteristic of the plasticization effect provided by ELO for plastic deformation, as aforementioned. It should be pointed out that modified vegetable oils are not completely miscible, since the addition of more than 5–7.5 wt% can lead to the increased presence of cavities, resulting in a worsening of the miscibility with a negative effect in ductile properties [48]. In Figure 4d, almost no presence of cavities was found (highlighted by yellow circle), which indicates that the incorporation of 5% ELO was highly miscible in both matrixes. For the Xibond incorporation, no significant differences in the morphology were observed regarding PHB75-PLA formulation, which is attributed to the similar absorbed energy values obtained in the Charpy test. As demonstrated in mechanical properties, Xibond did not improve the ductile properties.

#### 3.1.2. Thermal Properties

**Figure 5:**
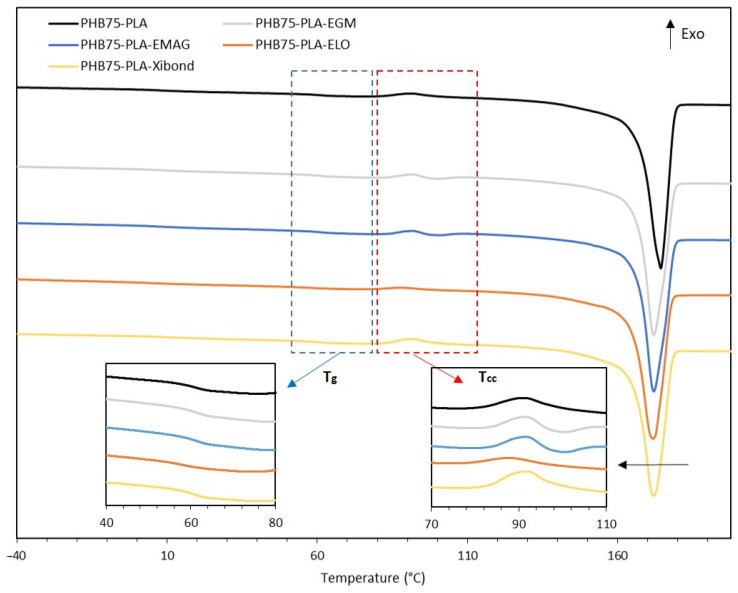
Differential scanning calorimetry (DSC) curves of PHB75-PLA blend composites. The black arrow in melt temperature region means a displacement to lower temperatures.

The thermal properties of PHB75-PLA composites with compatibilizer additives have been assessed by means of DSC and TGA analysis. The main thermal transition parameters from DSC analysis are summarized in Table 4 as well as in Figure 5. As expected, the glass transition temperature (T_g_) of PHB phase was not detected using DSC, as has been reported in the literature when pristine PHB phase was evaluated [49]. On the contrary, the T_g_ of the PLA phase in the PHB75-PLA sample was observed at a value of 60.6 °C. The addition of compatibilizers in the blend exhibited two different behaviours in the T_g_ of the PLA phase. The incorporation of EGM, EMAG and Xibond did not show significant differences. Nevertheless, the bio-based ELO shifted the T_g_ to lower temperatures from 60.6 to 58.0 °C for PHB75-PLA and PHB75-PLA-ELO, respectively. This is a typical plasticizing effect provided by modified vegetable oil that enhances the chain mobility within the sample and, therefore, decreasing the T_g_ [50]. The second thermal transition is related to the cold crystallization temperature (T_cc_), which was found to be 91.0 °C for the PHB75-PLA blend, and the incorporation of compatibilizers did not show any influence except for the bio-based ELO. As expected, the latter exhibited a shifted to lower temperatures caused by the more intense polymer chain motion when ELO is added [51]. The last thermal transition is related to the melt temperature (T_m_) of composites. For the PHB75-PLA sample, a single peak was observed at 174.3 °C due to the similar melting point of the employed polymers. In this regard, the compatibilizers decreased the T_m_ up to values in the range of 171.4 and 171.9 °C with no relevant changes between them in terms of shape and peak. Closely related to this, ΔH_m_ also exhibited a decrease with the additives, which indicates the formation of less-perfect crystals that ease its melting process [52]. Therefore, the crystallinity in all cases decreased by incorporating compatibilizers. For the samples compatibilized with EGM, EMAG and Xibond, a decrease from 61.2% for the PHB75-PLA sample to 55.3–56.9% was obtained, which suggests a formation of crosslinked, branch or chain extended structures within the polymer that makes the crystallization difficult. This effect was also observed with the addition of dycumil peroxide (DCP) in the partially crosslinked PHB/PDLLA blend [53]. This behaviour could be explained by the chemical interaction between the glycidyl, acrylate and maleated groups corresponding to EGM, EMAG and Xibond, respectively, with the hydroxyl and carboxyl groups of PHB and PLA hindering the crystal formation. On the other hand, it should be pointed out that the crystallinity of ELO decreased up to 58.7 °C but not as much as other compatibilizers. It can be ascribed to its known dual plasticizer–compatibilizer effect that not only leads to the improvement of the chain mobility but also the formation of branching structures. The same behaviour was reported by Garcia-Garcia et al. [54], where the use of ELO in the PHB matrix leads to a decrease in its crystallinity as well as its T_g_, due to the plasticizing effect of vegetable oils. It is worth noting that this decrease in crystallinity also provides advantages during 3D printing process. For instance, the reduction in crystallinity, which is also related to the shifting of T_m_ to lower temperatures, can improve the processability as well as employ less energy during the processing [55]. Besides, it is known that crystallinity plays a key role during 3D printing since it is directly linked to the shrinkage, promoting the detachment of the manufactured samples due to the warping effect [56]. In this sense, the addition of these additives suggests that they can reduce this effect, which is commonly found in semicrystalline polymers such as PHB and PLA. Furthermore, the crystallinity also affects the hydrolysis degradation process, which is an important point in bone tissue engineering. According to Feng et al. [57], the less-packed molecules (lower crystallinity), which accelerate the degradation process by means of the hydrolytic breakage of molecules due to water, can diffuse more easily into the matrix. Therefore, considering the aforementioned results, the additives studied could accelerate the degradation rate, since they showed a decrease in crystallinity compared with the pristine PHB/PLA.

**Figure 6:**
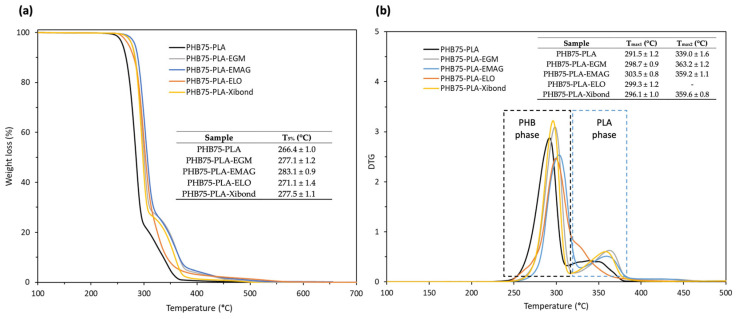
Thermal stability of PHB75-PLA blend with different additives: (**a**) thermogravimetric analysis (TGA) and (**b**) first-derivate thermogravimetric (DTG) curves.

The thermal degradation of PHB75-PLA blends with compatibilizers is shown in Figure 6. The pristine PHB75-PLA was thermally degraded in a two-step process, as was expected due to the binary blend. The first degradation process corresponds to PHB located from 5% up to 75% of weight loss due to a random chain scission thermally induced by the cleavage of both C-O and C=O bonds [58]. The second step is referred to degradation of PLA by chain-end scission through the breakage of ester groups [59], showing higher thermal stability compared to PHB. Concerning the temperature required for 5 wt% of weight loss (T_5%_) of the PHB75-PLA sample, it was observed at a value of 266.4 °C, which is in the range of both pristine polymers and nearest to the T_5%_ of PHB, since it is the PHB-rich phase. In general, the addition of all the studied compatibilizers leads to an increase in T_5%_ between 5 and 16 °C compared to the pristine PHB75-PLA, as is observed in Figure 6a. This indicates an effective stabilizing effect occurring in the formulations. Besides, the maximum temperature has been determined (T_max_) by the first derivate of weight loss, showing two characteristic peaks belonging to the PHB75-PLA blend, shown in Figure 6b. In this regard, the T_max1_ and T_max2_ refer to the PHB and PLA matrices, with values of 291.5 and 339 °C, respectively. These curves are in agreement with Arrieta et al. [60], who evaluated both matrices in different proportions by means of electrospinning technique. With regard to the addition of compatibilizers, a similar tendency towards higher temperatures was observed as aforementioned, achieving a noticeable improvement with values in the range of 296–303 °C and 359–363 °C compared to the pristine, which were 291.5 °C and 339 °C for T_max1_ and T_max2_, respectively. This behaviour can probably be ascribed to two main reasons. The first could be explained by the higher thermal stability of additives added, which are EGM [40], EGMA [61], ELO [62] and the styrene-based copolymer Xibond [38], which can contribute to improve the T_max1_ thermal stability referred to in the PHB-rich phase. The other fact is related to the interaction between the compatibilizers and the polymer matrices, which can positively affect the whole thermal stability. This increment suggests the reactivity between end-chain groups in both PLA and PHB (-COOH and -OH) with the corresponding reactive groups of compatibilizers such as glycidyl, acrylate and maleated groups. This effect was also reported by Abdelwahab et al. [63], who observed an increase in thermal stability in the PLA/PBAT compatibilization with a chain extender that contains glycidyl groups. Nevertheless, the T_max2_ of the bio-based ELO was not clearly shown as a result of the displacement to lower temperatures overlapping with the PHB-phase thermal degradation. According to Quiles-Carillo et al. [44], the incorporation of ELO into polymer blends leads to a decrease in the thermal stability caused by the increment of free volume for its plasticizer effect. Similar findings have been reported by other authors by adding functionalized vegetable oils such as soybean oil in order to improve the interaction between polymer blends [26]. In this manner, the enhancement of thermal degradation with the incorporation of additives leads to avoiding the possible thermal degradation involved during the manufacturing process, since, according to Caputo et al. [64], the degradation takes places even during processing at lower temperatures.

### 3.2. Scaffold Characterization

With the aim to assess the properties of 3D printed scaffolds with the different developed formulations, two geometries were manufactured, shown in Figure 2b,c. The printing parameters of the scaffolds were included in Table 2.

#### 3.2.1. Porosity, Pore Size and Surface Morphology

**Figure 7:**
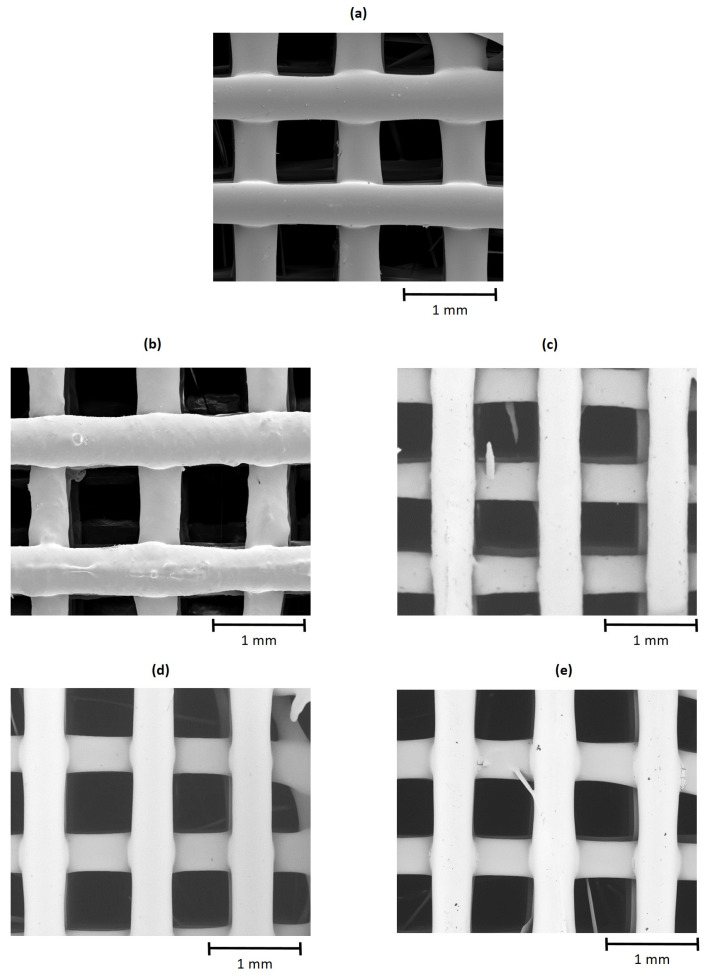
SEM images of 3D printed scaffolds of PHB75-PLA blend samples with additives: (**a**) PHB75-PLA, (**b**) PHB75-PLA-EGM, (**c**) PHB75-PLA-EMAG, (**d**) PHB75-PLA-ELO and (**e**) PHB75-PLA-Xibond.

The porosity and pore size of 3D printed scaffolds for the cell proliferation test are shown in Table 5. The bulk density of the pristine blend PHB75-PLA was 1.24 cm^3^, which are in the range of both matrices according to the datasheet of products. As expected, the incorporation of the compatibilizers did not modify this value, since they were not ceramic-filler type that increase the bulk density [65]. Regarding the porosity, which is a coefficient between material and apparent density, it was not changed regardless of the compatibilizers that were employed. The results indicate that all groups of scaffolds were printed with around 50% of porosity, which is considered suitable for the growth of bone cells [66]. In addition, the pore size, measured as the distance between filaments extruded, was also in the range of 675–718 μm, as shown in Figure 7. According to Karageorgiou and Kaplan [67], pores higher than 300 μm are required to promote new bone formation. Besides, other authors indicate that pore size of 300 and 800 μm did not affect this property, enhancing the bone in-growth [68]. As a result, the printed PHB75-PLA scaffolds demonstrated both favourable porosity and pore size for bone regeneration.

#### 3.2.2. Compression Test

**Figure 8:**
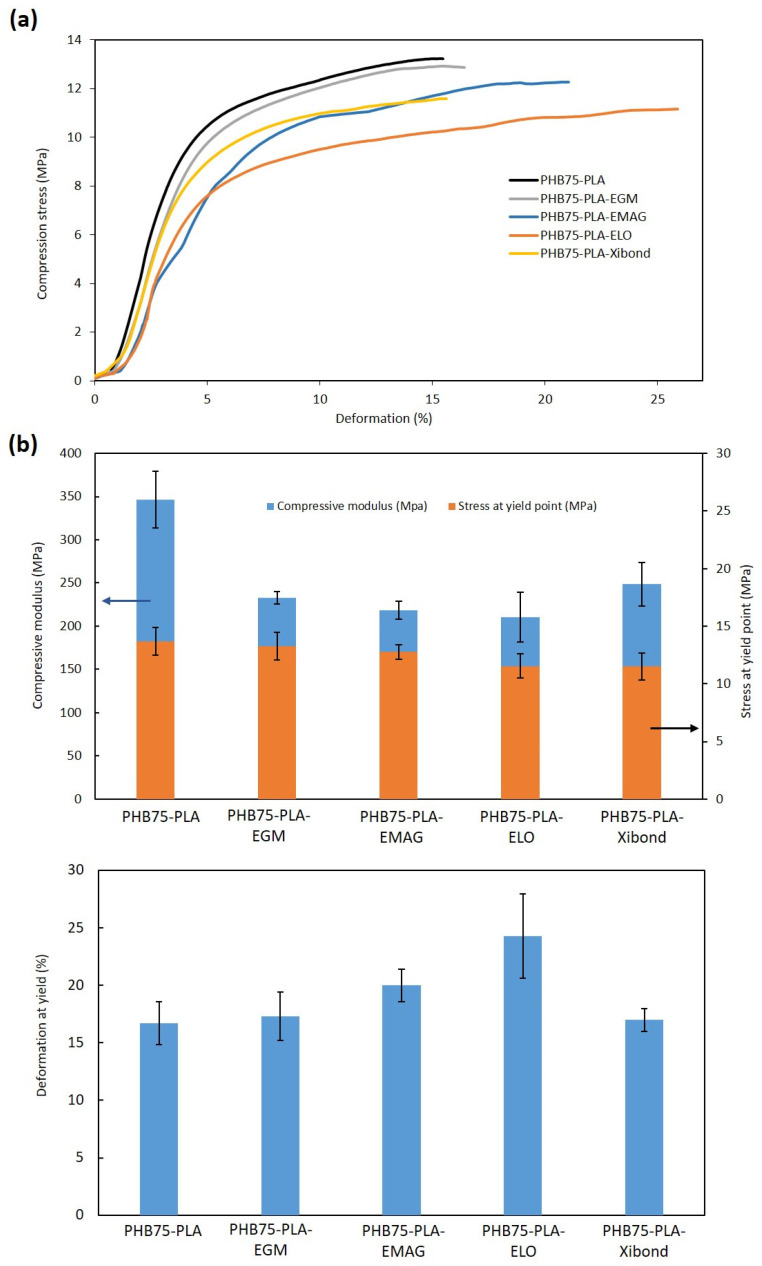
Compression test of 3D printed scaffolds of the PHB75-PLA blend with different additives: (**a**) stress–strain curves and (**b**) compression properties, where the blue arrow indicates compressive modulus and the black arrow indicates stress at yield.

Concerning the compression test, the samples exhibited a compression in the stress–strain graph, which corresponds to the linear elastic region (compression modulus) and stress–strain slope increasing up to the yield point to obtain stress and deformation. Both stress–strain curves and properties are shown in Figure 8. The compressive modulus of PHB75-PLA sample recorded a value of 346.6 MPa, whereas the incorporation of compatibilizers decreased it up to values of 210–250 MPa. The lowest modulus was obtained by additivation with ELO, which can be ascribed to its plasticizer effect, as reported by Alam et al. [69]. On the contrary, the highest modulus from compatibilized samples was reached with the incorporation of Xibond, suggesting the aforementioned compatibilizer effect. Despite the decrease in the compression modulus, all developed formulations showed higher values than needed in trabecular bones (50–100 MPa) [70] with an average improvement of 132%. Regarding the compressive stress at yield, no significant difference was observed in the compatibilized samples. However, their values are still in the range of 11–13 MPa that is required for trabecular bones (5–10 MPa) [71]. The deformation at yield showed values similar than the pristine PHB75-PLA sample (16.5%) except for EMAG and ELO with values around 20 and 23%, respectively. Comparing these values with the developed scaffolds in the literature, a similar behaviour was observed with regard to printed scaffolds with a porosity close to 70% of the pristine P(3HB-co-3HHx) and with the addition of HA [33]. In the present work, the deformation at yield point was higher due to the incorporation of specific additives, whereas the incorporation of HA from 2.5% to 10% in the aforementioned paper led to a decrease in this property, as expected, from 18 to 16%, respectively.

#### 3.2.3. Water Contact Angle Measurement (WCA)

**Figure 9:**
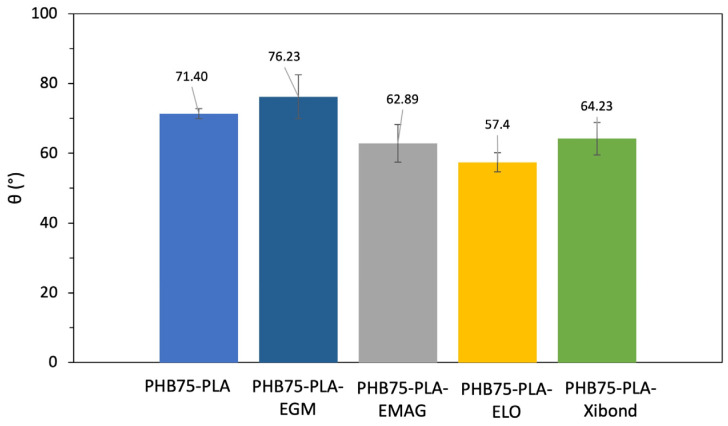
Water contact angle measurements of the 3D printed scaffolds of the PHB75-PLA blend with different additives.

The hydrophilic behaviour of samples has been assessed by static water contact of flat surfaces manufactured by MEX. The WCA obtained for the different developed formulations are shown in Figure 9. The pristine blend PHB75-PLA showed a WCA of 71.4°, which is considered a hydrophobic surface (θ < 65°) [72]. These results are in concordance with values reported in the literature, which is quite similar to the pristine PHB [73]. Nevertheless, the incorporation of the compatibilizers changed the surface wettability. For the polyethylene derivatives, the sample PHB75-PLA-EGM did not modify the wetting properties compared to PHB75-PLA, whereas the incorporation of the acrylate group, which is the sample PHB75-PLA-EMAG, enhanced the wettability, reaching values of 62.9° and then caused a hydrophilic behaviour. In the case of the addition of the bio-based compatibilizer ELO, a decrease in WCA from 71.40° for PHB75-PLA to 57.4° was observed. This improvement of the hydrophilic surface has been also observed using epoxidized soybean oil (ESBO) in a PLA matrix [74]. Regarding the incorporation of Xibond as a compatibilizer, a similar trend was observed for ELO with a value of 64.23°. The increase in wetting properties can be ascribed to the polar groups within the chemical structure of Xibond, such as glycidil and maleic anhydride functionalities [33], increasing the capability to interact with other surrounding molecules. Therefore, the addition of EMAG, ELO and Xibond compatibilizers provides a change from hydrophobic to hydrophilic surfaces (θ < 65°), which can be a positive effect in cell attachment.

#### 3.2.4. Cell Metabolic Activity Evaluation

**Figure 10:**
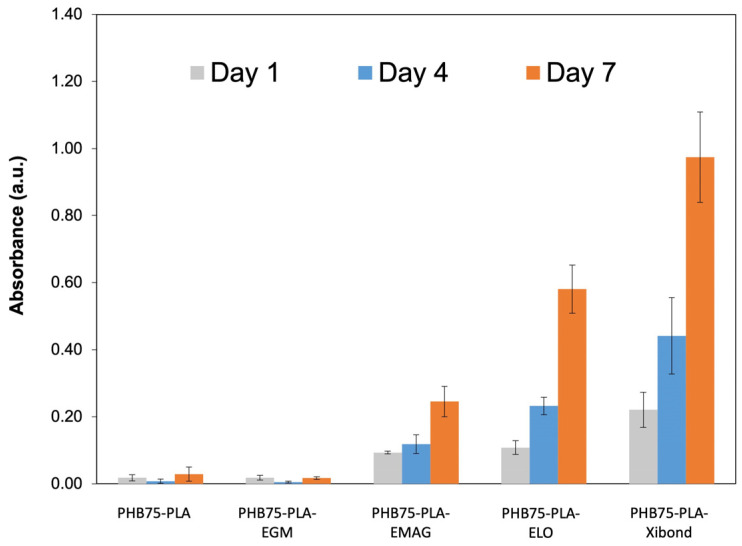
Metabolic activity of human foetal osteoblastic (hFOB) cells of printed scaffolds.

The cell metabolic activity of the printed scaffolds was evaluated using a CCK-8 assay with human foetal osteoblastic (hFOB) cells, for which absorbance values correlate to the number of cells. In Figure 10, the absorbance values are shown from printed scaffolds with different additives. Both the pristine PHB75-PLA and PHB75-PLA-EGM samples did not show an increment of absorbance, which indicates that seeded cells did not attach on the surface during 7 days of culture. It can be attributed to the hydrophilic behaviour of samples since they are the highest assessed (76–71°). According to Lim et al. [75], the surface wettability plays a key role on cell attachment, thus hydrophobic surfaces (θ < 65°) result in a lesser growth of cells. In this regard, the EMAG, ELO and Xibond additives with a WCA in a range of 64–57° provide an increment of bone cells attached on the surface that proliferate from day 1 to 7, likely for its better wettability. The hydrophilic surfaces formulated improve the initial protein interactions, the osteoblast cell adhesion and its spreading. Concerning the difference in cell metabolic activity between formulations with similar WCA, which are EMAG, ELO and Xibond formulations, it should be mentioned that chemical groups of formulations can also affect cell proliferation, as has been reported [76]. Based on the results, it is suggested that maleated and phenyl groups contained in a Xibond additive lead to favour the cell adhesion on the surface compared with the epoxy and glycidyl groups of ELO and EMAG, respectively. In addition, all chemical groups that are found in the additives incorporated in the PHB/PLA blend are considered non-toxic according to the literature. For instance, Ibrahim et al. [77] characterized hydrogel scaffold crosslinked with glycidyl methacrylate, a group found in the EGM and EMAG additive, and showed good cell proliferation with no cytotoxicity. Similar behaviour has been reported by Miao Shida et al. [76], where soybean oil epoxidized acrylate, whose chemical structure is very similar to ELO, only different by the number of epoxy groups and also presenting an acrylate group (found in the EMAG additive), was assessed as a non-toxic substance with cell-proliferation values similar to PLA or PCL. Finally, both maleated and phenyl groups found in the Xibond additive have also been assessed in other studies. Zhou et al. [78] reported that PLA grafted with maleic anhydride did not exhibit cytotoxicity in cellular viability, whereas the phenyl group is found in the acrylonitrile butadiene styrene (ABS) structure, which is commonly used for biomedical applications [79]. However, with the aim to precisely corroborate its cytotoxicity, a specific test should be made in further studies.

**Figure 11:**
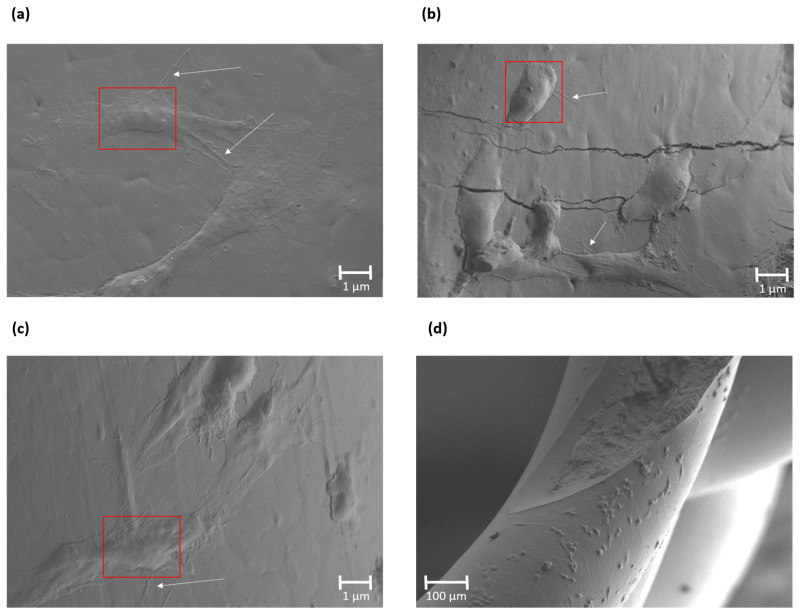
SEM images of cell proliferation of human osteoblastic cells on 3D printed scaffolds after 7 days: (**a**) PHB75-PLA-EMAG (500×); (**b**) PHB75-PLA-ELO (500×); (**c**) PHB75-PLA-Xibond (500×); (**d**) PHB75-PLA-Xibond (125×). Red box indicates lamellipodia and white arrow a filopodia formation.

The SEM images of the scaffolds that exhibited cell proliferation after 7 days of culture are shown in Figure 11. The scaffold’s interior with the incorporation of EMAG, ELO and Xibond compatibilizers showed both filopodia and lamellipodia cytoplasmic processes on the surface. In the mentioned samples, the cells exhibited a flatter and widespread adhesion on the surface by lamellipodia formation with a branched network, as highlighted in red box. This indicates stable attachments on the surface guided by filopodia, marked with a white arrow, in order to promote cell mobility [80]. The presence of these filopodia indicates a cell–cell interaction that can provide new lamellipodia nucleation [81]. In this sense, all samples showed cell growth with a more extended grade in the case of the PHB75-PLA-Xibond sample, as can be observed in Figure 11d, indicating its potential to be applied in bone regeneration applications.

## 4. Conclusions

This work showed the effect of additives in the cell metabolic activity, and other mechanical and thermal properties of a PHB-rich phase and PLA blend (75/25 wt%) formulations to manufacture scaffolds for tissue engineering. The compounding and processability of the formulations were also studied. In the first stage, the formulations were assessed to evaluate the interaction between additives and the polymer matrix blend using standard geometries manufactured by MEX. The flexural test showed that EGM and EMAG slightly increased the strain with their consequent decrease in modulus compared to the pristine blend. The highest compression strain was obtained using bio-based ELO with an enhancement of 32% due its plasticizing effect, whereas Xibond provided the highest flexural strength with an increment of 6.5% regarding PHB75-PLA. For this reason, the Charpy test showed that EGM, EMAG and bio-based ELO led to an increase in the absorbed energy in the high strain rate test, with improvements between 31 and 47%, as observed by the trend in flexural stress. This behaviour was corroborated by the fractured morphology, showing a more pronounce rough surface for the addition of EGM, EMAG and ELO. Regarding the thermal properties, the T_g_ of the blend was reduced by 2.6 °C with the addition of bio-based ELO due to the increment of free volume that enhanced the chain mobility. Besides, all formulations with the additives showed a decrease between 2.1 and 2.9 °C of T_m_ with respect to the PHB75-PLA blend. This improvement, coupled with the shift from 291.5 °C and 339 °C to 296–303 °C and 359–363 °C in T_max1_ and T_max2_, respectively, provided an enhancement of thermal stability to the blend that improved the processability during the MEX manufacturing, reducing the possible thermal degradation.

In the second stage, all 3D printed scaffolds exhibited a porosity and mean pore size in the range of 50.3–53.3% and 675–718 μm, respectively, which is adequate for being applied in tissue engineering. In addition, scaffolds with additives showed values higher (11–13 MPa) than the required for trabecular bones (5–10 MPa) in compression stress. On one hand, the bio-based ELO and EMAG showed an improvement of deformation at yield with values of 23 and 20%, respectively, compared to the pristine blend (16.5%). On the other hand, Xibond provided the highest compressive modulus between additives, remarking on its compatibilizing effect. Different results were obtained regarding the cell metabolic activity of the scaffolds. The incorporation of EMAG and, to a greater extent, the bio-based ELO and Xibond additives, led to an increase in the cell attachment on the surface. The wettability of the surface highly influences these values. Thus, the incorporation of the latter additives decreases the WCA to less than θ < 65°, providing a hydrophilic surface with a better cell adhesion. The cells showed stable attachment on the surface after 7 days of culture with good shape of both filopodia and lamellipodia processes. Therefore, according to the results, both the EMAG and bio-based ELO provided greater ductile properties to the scaffolds, highlighting ELO in terms of cell metabolic activity. In contrast, Xibond showed a higher compressive modulus with the highest cell proliferation. These results show the potential of these scaffolds with additives for bone regeneration, highlighting their possible use coupled with inorganic fillers that can promote a higher capability of cell proliferation.

In view of the results, it is suggested that further studies focus on different aspects that could not be addressed in the current paper. The cytotoxicity of the scaffolds is one of the main aspects that should be carried out in order to corroborate the biocompatibility of added additives. From a mechanical point of view, immersion of scaffolds in Phosphate Buffered Saline (PBS) is required to assess the hydrolytic degradation as well as the dynamic fatigue testing that can provide valuable information in addressing bone regeneration applications.

## Figures and Tables

**Table 1:** Composition of PHB/PLA biocomposites.

			Parts Per Hundred Resin (phr)			
Sample Code	PHB (wt%)	PLA (wt%)	EGM	EMAG	ELO	Xibond
PHB75-PLA	75	25	-	-	-	-
PHB75-PLA-EGM	75	25	5	-	-	-
PHB75-PLA-EMAG	75	25	-	5	-	-
PHB75-PLA-ELO	75	25	-	-	5	-
PHB75-PLA-Xibond	75	25	-	-	-	5

**Table 2:** Printing parameters for the standards geometries and scaffolds.

Printing Parameters	Standards Geometries	Scaffolds
Nozzle (mm)	0.4	0.4
Printing temperature (°C)	195	195
Bed temperature (°C)	45	45
Printing speed (mm/s)	50	15
Layer height (mm)	0.2	0.2
Infill (%)	100	30
Raster angle (°)	0/90	0/90

**Table 3:** Flexural test of non-porous PHB75-PLA blend samples with additives.

Sample	Flexural Modulus (MPa)	Flexural Strength (MPa)	Flexural Strain (%)
PHB75-PLA	3330 ± 120	53.5 ± 4.0	3.1 ± 0.3
PHB75-PLA-EGM	2900 ± 254	52.5 ± 1.7	3.4 ± 0.3
PHB75-PLA-EMAG	3050 ± 124	52.6 ± 4.7	3.7 ± 0.3
PHB75-PLA-ELO	3080 ± 207	54.1 ± 1.3	4.6 ± 0.2
PHB75-PLA-Xibond	3200 ± 135	57.0 ± 1.6	3.3 ± 0.4

**Table 4:** Differential scanning calorimetry (DSC) thermal properties of PHB75-PLA blend composites.

Sample	T_g_ (°C)	ΔH_cc_ (J·g^−1^)	T_cc_ (°C)	ΔH_m_ (J·g^−1^)	T_m_ (°C)	X_c_ (%)
PHB75-PLA	60.6 ± 0.6	1.51 ± 0.1	91.0 ± 0.4	82.8 ± 1.2	174.3 ± 0.5	61.2 ± 0.5
PHB75-PLA-EGM	61.0 ± 0.4	1.64 ± 0.2	91.7 ± 0.5	76.8 ± 1.0	171.4 ± 0.4	56.6 ± 0.4
PHB75-PLA-EMAG	61.5 ± 0.5	1.69 ± 0.1	91.2 ± 0.5	75.1 ± 0.9	172.2 ± 0.6	55.3 ± 0.4
PHB75-PLA-ELO	58.0 ± 0.7	0.82 ± 0.1	87.8 ± 0.6	78.9 ± 1.1	171.5 ± 0.4	58.7 ± 0.3
PHB75-PLA-Xibond	60.9 ± 0.6	3.08 ± 0.3	91.4 ± 0.8	78.6 ± 0.9	171.9 ± 0.5	56.9 ± 0.5

**Table 5:** Porosity and pore size of 3D printed scaffolds of the PHB75-PLA blend with different additives.

Sample	Bulk Density (g/cm^3^)	Apparent Density (g/cm^3^)	Porosity (%)	Pore Size (μm)
PHB75-PLA	1.24 ± 0.01	0.57 ± 0.04	53.3 ± 3.7	710.7 ± 28.8
PHB75-PLA-EGM	1.20 ± 0.02	0.57 ± 0.03	52.5 ± 3.8	718.4 ± 40.5
PHB75-PLA-EMAG	1.21 ± 0.02	0.57 ± 0.01	52.9 ± 1.5	675.0 ± 25.0
PHB75-PLA-ELO	1.22 ± 0.02	0.60 ± 0.02	50.3 ± 2.1	712.3 ± 37.5
PHB75-PLA-Xibond	1.22 ± 0.01	0.58 ± 0.03	52.2 ± 3.5	696.5 ± 18.2

## Data Availability

The data presented in this study are available on request from the author.

## References

[1] Vigil Fuentes M.A., Thakur S., Wu F., Misra M., Gregori S., Mohanty A.K. (2020). Study on the 3D Printability of Poly(3-Hydroxybutyrate-Co-3-Hydroxyvalerate)/Poly(Lactic Acid) Blends with Chain Extender Using Fused Filament Fabrication. Sci. Rep..

[2] Gibson I., Rosen D.W., Stucker B., Khorasani M., Rosen D., Stucker B., Khorasani M. (2021). Additive Manufacturing Technologies.

[3] Wang S., Li R., Qing Y., Wei Y., Wang Q., Zhang T., Sun C., Qin Y., Li D., Yu J. (2019). Antibacterial Activity of Ag-Incorporated Zincosilicate Zeolite Scaffolds Fabricated by Additive Manufacturing. Inorg. Chem. Commun..

[4] Abbasi N., Hamlet S., Love R.M., Nguyen N.-T. (2020). Porous Scaffolds for Bone Regeneration. J. Sci. Adv. Mater. Devices.

[5] Gregor A., Filová E., Novák M., Kronek J., Chlup H., Buzgo M., Blahnová V., Lukášová V., Bartoš M., Nečas A. (2017). Designing of PLA Scaffolds for Bone Tissue Replacement Fabricated by Ordinary Commercial 3D Printer. J. Biol. Eng..

[6] Laschke M.W., Augustin V.A., Sahin F., Anschütz D., Metzger W., Scheuer C., Bischoff M., Aktas C., Menger M.D. (2016). Surface Modification by Plasma Etching Impairs Early Vascularization and Tissue Incorporation of Porous Polyethylene (Medpor^®^) Implants. J. Biomed. Mater. Res. B Appl. Biomater..

[7] Rebia R.A., Rozet S., Tamada Y., Tanaka T. (2018). Biodegradable PHBH/PVA Blend Nanofibers: Fabrication, Characterization, in Vitro Degradation, and in Vitro Biocompatibility. Polym. Degrad. Stab..

[8] Tajvar S., Hadjizadeh A., Samandari S.S. (2023). Scaffold Degradation in Bone Tissue Engineering: An Overview. Int. Biodeterior. Biodegrad..

[9] Wang W., Caetano G., Ambler W.S., Blaker J.J., Frade M.A., Mandal P., Diver C., Bártolo P. (2016). Enhancing the Hydrophilicity and Cell Attachment of 3D Printed PCL/Graphene Scaffolds for Bone Tissue Engineering. Materials.

[10] Sabino M., Fermín Z., Marielys L., Moret J., Rodríguez D., Rezende R.A., Neto P.I., Pereira F.D.S., Silva J.V.L., Alvarez J. (2013). In Vitro Biocompatibility Study of Biodegradable Polyester Scaffolds Constructed Using Fused Deposition Modeling (FDM). IFAC Proc. Vol..

[11] Tyler B., Gullotti D., Mangraviti A., Utsuki T., Brem H. (2016). Polylactic Acid (PLA) Controlled Delivery Carriers for Biomedical Applications. Adv. Drug Deliv. Rev..

[12] Daculsi G., Goyenvalle E., Cognet R., Aguado E., Suokas E.O. (2011). Osteoconductive Properties of Poly(96L/4D-Lactide)/Beta-Tricalcium Phosphate in Long Term Animal Model. Biomaterials.

[13] Abert J., Amella A., Weigelt S., Fischer H. (2016). Degradation and Swelling Issues of Poly-(d,l-Lactide)/β-Tricalcium Phosphate/Calcium Carbonate Composites for Bone Replacement. J. Mech. Behav. Biomed. Mater..

[14] Qu X.-H., Wu Q., Zhang K.-Y., Chen G.Q. (2006). In Vivo Studies of Poly(3-Hydroxybutyrate-Co-3-Hydroxyhexanoate) Based Polymers: Biodegradation and Tissue Reactions. Biomaterials.

[15] Kontárová S., Přikryl R., Melčová V., Menčík P., Horálek M., Figalla S., Plavec R., Feranc J., Sadílek J., Pospíšilová A. (2020). Printability, Mechanical and Thermal Properties of Poly(3-Hydroxybutyrate)-Poly(Lactic Acid)-Plasticizer Blends for Three-Dimensional (3D) Printing. Materials.

[16] Zhuikov V.A., Akoulina E.A., Chesnokova D.V., Wenhao Y., Makhina T.K., Demyanova I.V., Zhuikova Y.V., Voinova V.V., Belishev N.V., Surmenev R.A. (2021). The Growth of 3T3 Fibroblasts on PHB, PLA and PHB/PLA Blend Films at Different Stages of Their Biodegradation In Vitro. Polymers.

[17] Arrieta M.P., Samper M.D., Aldas M., López J. (2017). On the Use of PLA-PHB Blends for Sustainable Food Packaging Applications. Materials.

[18] Mokrane N., Kaci M., Lopez-Cuesta J.-M., Dehouche N. (2023). Combined Effect of Poly(Lactic Acid)-Grafted Maleic Anhydride Compatibilizer and Halloysite Nanotubes on Morphology and Properties of Polylactide/Poly(3-Hydroxybutyrate-Co-3-Hydroxyhexanoate) Blends. Materials.

[19] Frone A.N., Batalu D., Chiulan I., Oprea M., Gabor A.R., Nicolae C.-A., Raditoiu V., Trusca R., Panaitescu D.M. (2020). Morpho-Structural, Thermal and Mechanical Properties of PLA/PHB/Cellulose Biodegradable Nanocomposites Obtained by Compression Molding, Extrusion, and 3D Printing. Nanomaterials.

[20] Montava-Jordà S., Quiles-Carrillo L., Richart N., Torres-Giner S., Montanes N. (2019). Enhanced Interfacial Adhesion of Polylactide/Poly(ε-Caprolactone)/Walnut Shell Flour Composites by Reactive Extrusion with Maleinized Linseed Oil. Polymers.

[21] Lopera-Valle A., Caputo J.V., Leão R., Sauvageau D., Luz S.M., Elias A. (2019). Influence of Epoxidized Canola Oil (ECO) and Cellulose Nanocrystals (CNCs) on the Mechanical and Thermal Properties of Polyhydroxybutyrate (PHB)—Poly(Lactic Acid) (PLA) Blends. Polymers.

[22] Garcia-Campo M.J., Quiles-Carrillo L., Sanchez-Nacher L., Balart R., Montanes N. (2019). High Toughness Poly(Lactic Acid) (PLA) Formulations Obtained by Ternary Blends with Poly(3-Hydroxybutyrate) (PHB) and Flexible Polyesters from Succinic Acid. Polym. Bull..

[23] Xiao X., Chevali V.S., Song P., He D., Wang H. (2019). Polylactide/Hemp Hurd Biocomposites as Sustainable 3D Printing Feedstock. Compos. Sci. Technol..

[24] Jorda M., Montava-Jorda S., Balart R., Lascano D., Montanes N., Quiles-Carrillo L. (2019). Functionalization of Partially Bio-Based Poly(Ethylene Terephthalate) by Blending with Fully Bio-Based Poly(Amide) 10,10 and a Glycidyl Methacrylate-Based Compatibilizer. Polymers.

[25] Bin Y., Yang B., Wang H. (2018). The Effect of a Small Amount of Modified Microfibrillated Cellulose and Ethylene–Glycidyl Methacrylate Copolymer on the Crystallization Behaviors and Mechanical Properties of Polylactic Acid. Polym. Bull..

[26] Garcia-Campo M.J., Quiles-Carrillo L., Masia J., Reig-Pérez M.J., Montanes N., Balart R. (2017). Environmentally Friendly Compatibilizers from Soybean Oil for Ternary Blends of Poly(Lactic Acid)-PLA, Poly(ε-Caprolactone)-PCL and Poly(3-Hydroxybutyrate)-PHB. Materials.

[27] Kilic N.T., Can B.N., Kodal M., Ozkoc G. (2019). Compatibilization of PLA/PBAT Blends by Using Epoxy-POSS. J. Appl. Polym. Sci..

[28] Vernaez O., Neubert K.J., Kopitzky R., Kabasci S. (2019). Compatibility of Chitosan in Polymer Blends by Chemical Modification Of-Based Polyesters. Polymers.

[29] (2019). Plastics—Determination of Tensile Propertiespart 1: General Principles.

[30] Puppi D., Chiellini F., Piras A.M., Chiellini E. (2010). Polymeric Materials for Bone and Cartilage Repair. Prog. Polym. Sci..

[31] (2019). Plastics–Determination of Flexural Properties.

[32] (2003). UNE-EN-ISO, UNE Norma Española. 604. Plásticos, Determinación de Las Propiedades En Compresión.

[33] Ivorra-Martinez J., Ferrer I., Aguado R., Delgado-Aguilar M., Garcia-Romeu M.L., Boronat T. (2024). Development of P (3HB-Co-3HHx) Nanohydroxyapatite (NHA) Composites for Scaffolds Manufacturing by Means of Fused Deposition Modeling. Int. J. Bioprint..

[34] Loureiro N.C., Ghosh S., Viana J.C., Esteves J.L. (2015). Thermal Characterization of Polyhydroxyalkanoates and Poly(Lactic Acid) Blends Obtained by Injection Molding. Polym. Plast. Technol. Eng..

[35] Meng B., Deng J., Liu Q., Wu Z., Yang W. (2012). Transparent and Ductile Poly(Lactic Acid)/Poly(Butyl Acrylate) (PBA) Blends: Structure and Properties. Eur. Polym. J..

[36] Maziad N.A., EL-Nashar D.E., Sadek E.M. (2009). The Effects of a Silane Coupling Agent on Properties of Rice Husk-Filled Maleic Acid Anhydride Compatibilized Natural Rubber/Low-Density Polyethylene Blend. J. Mater. Sci..

[37] Yuan Y., Lee T.R. (2013). Contact Angle and Wetting Properties. Surface Science Techniques.

[38] Donate R., Monzón M., Ortega Z., Wang L., Ribeiro V., Pestana D., Oliveira J.M., Reis R.L. (2020). Comparison between Calcium Carbonate and β-Tricalcium Phosphate as Additives of 3D Printed Scaffolds with Polylactic Acid Matrix. J. Tissue Eng. Regen. Med..

[39] Tejada-Oliveros R., Gomez-Caturla J., Sanchez-Nacher L., Montanes N., Quiles-Carrillo L. (2021). Improved Toughness of Polylactide by Binary Blends with Polycarbonate with Glycidyl and Maleic Anhydride-Based Compatibilizers. Macromol. Mater. Eng..

[40] Chang B.P., Mohanty A.K., Misra M. (2018). Tuning the Compatibility to Achieve Toughened Biobased Poly(Lactic Acid)/Poly(Butylene Terephthalate) Blends. RSC Adv..

[41] Dominguez-Candela I., Gomez-Caturla J., Cardona S.C., Lora-García J., Fombuena V. (2022). Novel Compatibilizers and Plasticizers Developed from Epoxidized and Maleinized Chia Oil in Composites Based on PLA and Chia Seed Flour. Eur. Polym. J..

[42] Han Y., Shi J., Mao L., Wang Z., Zhang L. (2020). Improvement of Compatibility and Mechanical Performances of PLA/PBAT Composites with Epoxidized Soybean Oil as Compatibilizer. Ind. Eng. Chem. Res..

[43] Graupner N., Müssig J. (2011). A Comparison of the Mechanical Characteristics of Kenaf and Lyocell Fibre Reinforced Poly(Lactic Acid) (PLA) and Poly(3-Hydroxybutyrate) (PHB) Composites. Compos. Part A Appl. Sci. Manuf..

[44] Quiles-Carrillo L., Fenollar O., Balart R., Torres-Giner S., Rallini M., Dominici F., Torre L. (2020). A Comparative Study on the Reactive Compatibilization of Melt-Processed Polyamide 1010/Polylactide Blends by Multi-Functionalized Additives Derived from Linseed Oil and Petroleum. Express Polym. Lett..

[45] Iglesias-Montes M.L., Soccio M., Siracusa V., Gazzano M., Lotti N., Cyras V.P., Manfredi L.B. (2022). Chitin Nanocomposite Based on Plasticized Poly(Lactic Acid)/Poly(3-Hydroxybutyrate) (PLA/PHB) Blends as Fully Biodegradable Packaging Materials. Polymers.

[46] D’Amico D.A., Iglesias Montes M.L., Manfredi L.B., Cyras V.P. (2016). Fully Bio-Based and Biodegradable Polylactic Acid/Poly(3-Hydroxybutirate) Blends: Use of a Common Plasticizer as Performance Improvement Strategy. Polym. Test.

[47] Sui G., Wang K., Xu S., Liu Z., Zhang Q., Du R., Fu Q. (2019). The Combined Effect of Reactive and High-Shear Extrusion on the Phase Morphologies and Properties of PLA/OBC/EGMA Ternary Blends. Polymer.

[48] Perez-Nakai A., Lerma-Canto A., Dominguez-Candela I., Ferri J.M., Fombuena V. (2023). Novel Epoxidized Brazil Nut Oil as a Promising Plasticizing Agent for PLA. Polymers.

[49] Mohapatra S., Sarkar B., Samantaray D.P., Daware A., Maity S., Pattnaik S., Bhattacharjee S. (2017). Bioconversion of Fish Solid Waste into PHB Using Bacillus Subtilis Based Submerged Fermentation Process. Environ. Technol..

[50] Dominguez-Candela I., Ferri J.M., Cardona S.C., Lora J., Fombuena V. (2021). Dual Plasticizer/Thermal Stabilizer Effect of Epoxidized Chia Seed Oil (*Salvia hispanica* L.) to Improve Ductility and Thermal Properties of Poly(Lactic Acid). Polymers.

[51] Balart J.F., Fombuena V., Fenollar O., Boronat T., Sánchez-Nacher L. (2016). Processing and Characterization of High Environmental Efficiency Composites Based on PLA and Hazelnut Shell Flour (HSF) with Biobased Plasticizers Derived from Epoxidized Linseed Oil (ELO). Compos. B Eng..

[52] Lai S.-M., Li H.-C., Liao Y.-C. (2007). Properties and Preparation of Compatibilized Nylon 6 Nanocomposites/ABS Blends: Part II—Physical and Thermal Properties. Eur. Polym. J..

[53] Dong W., Ma P., Wang S., Chen M., Cai X., Zhang Y. (2013). Effect of Partial Crosslinking on Morphology and Properties of the Poly(β-Hydroxybutyrate)/Poly(d,l-Lactic Acid) Blends. Polym. Degrad. Stab..

[54] Garcia-Garcia D., Ferri J.M., Montanes N., Lopez-Martinez J., Balart R. (2016). Plasticization Effects of Epoxidized Vegetable Oils on Mechanical Properties of Poly(3-Hydroxybutyrate). Polym. Int..

[55] Benwood C., Anstey A., Andrzejewski J., Misra M., Mohanty A.K. (2018). Improving the Impact Strength and Heat Resistance of 3D Printed Models: Structure, Property, and Processing Correlationships during Fused Modeling (FDM) of Poly(Lactic Acid). ACS Omega.

[56] Gonzalez-Gutierrez J., Cano S., Schuschnigg Stephan and Kukla C., Sapkota J., Holzer C. (2018). Additive Manufacturing of Metallic and Ceramic Components by the Material Extrusion of Highly-Filled Polymers: A Review and Future. Materials.

[57] Feng P., Jia J., Yu L., Min A., Yang S., Shuai C. (2021). Accelerated Degradation of Poly(L-Lactide) Bone Scaffold: Crystallinity Hydrophilicity. Mater. Chem. Phys..

[58] Hablot E., Bordes P., Pollet E., Avérous L. (2008). Thermal and Thermo-Mechanical Degradation of Poly(3-Hydroxybutyrate)-Based Multiphase Systems. Polym. Degrad. Stab..

[59] Sánchez-Jiménez P.E., Pérez-Maqueda L.A., Perejón A., Criado J.M. (2010). Generalized Kinetic Master Plots for the Thermal Degradation of Polymers Following a Random Scission Mechanism. J. Phys. Chem. A.

[60] Arrieta M.P., López J., López D., Kenny J.M., Peponi L. (2015). Development of Flexible Materials Based on Plasticized Electrospun PLA–PHB Blends: Structural, Thermal, Mechanical and Disintegration Properties. Eur. Polym. J..

[61] Yang Z., Zhou C., Cai J., Yan H., Huang X., Yang H., Cheng R. (2010). Effects of Macromolecular Compatibilizers Containing Epoxy Groups on the Properties of Linear Low-Density Polyethylene/Magnesium Hydroxide Composites. Ind. Eng. Chem. Res..

[62] Jebrane M., Cai S., Panov D., Yang X., Terziev N. (2015). Synthesis and Characterization of New Vinyl Acetate Grafting onto Epoxidized Linseed Oil in Aqueous Media. J. Appl. Polym. Sci..

[63] Abdelwahab M.A., Taylor S., Misra M., Mohanty A.K. (2015). Thermo-Mechanical Characterization of Bioblends from Polylactide and Poly(Butylene Adipate-Co-Terephthalate) and Lignin. Macromol. Mater. Eng..

[64] Caputo M.R., Fernandez M., Aguirresarobe Robert and Kovalcik A., Sardon H., Candal M.V., Mueller A.J. (2023). Influence of FFF Process Conditions on the Thermal, Mechanical, and Rheological Properties of Poly(Hydroxybutyrate-Co-Hydroxy Hexanoate). Polymers.

[65] Ivorra-Martinez J., Quiles-Carrillo L., Boronat T., Torres-Giner S., Covas J.A. (2020). Assessment of the Mechanical and Thermal Properties of Injection-Molded Poly(3-Hydroxybutyrate-Co-3-Hydroxyhexanoate)/Hydroxyapatite Nanoparticles Parts for Use in Bone Tissue Engineering. Polymers.

[66] McGregor M., Patel S., McLachlin S., Vlasea M. (2021). Architectural Bone Parameters and the Relationship to Titanium Lattice Design for Powder Bed Fusion Additive Manufacturing. Addit. Manuf..

[67] Karageorgiou V., Kaplan D. (2005). Porosity of 3D Biomaterial Scaffolds and Osteogenesis. Biomaterials.

[68] Roosa S.M.M., Kemppainen J.M., Moffitt E.N., Krebsbach P.H., Hollister S.J. (2010). The Pore Size of Polycaprolactone Scaffolds Has Limited Influence on Bone Regeneration in an in Vivo Model. J. Biomed. Mater. Res. A.

[69] Alam J., Alam M., Raja M., Abduljaleel Z., Dass L.A. (2014). MWCNTs-Reinforced Epoxidized Linseed Oil Plasticized Polylactic Acid Nanocomposite and Its Electroactive Shape Memory Behaviour. Int. J. Mol. Sci..

[70] Narayanan G., Vernekar V.N., Kuyinu E.L., Laurencin C.T. (2016). Poly (Lactic Acid)-Based Biomaterials for Orthopaedic Regenerative Engineering. Adv. Drug Deliv. Rev..

[71] Wubneh A., Tsekoura E.K., Ayranci C., Uludağ H. (2018). Current State of Fabrication Technologies and Materials for Bone Tissue Engineering. Acta Biomater..

[72] Vogler E.A. (1998). Structure and Reactivity of Water at Biomaterial Surfaces. Adv. Colloid Interface Sci..

[73] Senatov F., Zimina A., Chubrik A., Kolesnikov E., Permyakova E., Voronin A., Poponova M., Orlova P., Grunina T., Nikitin K. (2022). Effect of Recombinant BMP-2 and Erythropoietin on Osteogenic Properties of Biomimetic PLA/PCL/HA and PHB/HA Scaffolds in Critical-Size Cranial Defects Model. Biomater. Adv..

[74] Darie-Niţă R.N., Vasile C., Irimia A., Lipşa R., Râpă M. (2016). Evaluation of Some Eco-Friendly Plasticizers for PLA Films Processing. J. Appl. Polym. Sci..

[75] Lim J.Y., Taylor A.F., Li Z., Vogler E.A., Donahue H.J. (2005). Integrin Expression and Osteopontin Regulation in Human Fetal Osteoblastic Cells Mediated by Substratum Surface Characteristics. Tissue Eng..

[76] Miao S., Zhu W., Castro N.J., Nowicki M., Zhou X., Cui H., Fisher J.P., Zhang L.G. (2016). 4D Printing Smart Biomedical Scaffolds with Novel Soybean Oil Epoxidized Acrylate. Sci. Rep..

[77] Ibrahim S., Kothapalli C.R., Kang Q.K., Ramamurthi A. (2011). Characterization of Glycidyl Methacrylate—Crosslinked Hyaluronan Scaffolds Incorporating Elastogenic Hyaluronan Oligomers. Acta Biomater..

[78] Zhou C., Shi Q., Guo W., Terrell Lekeith and Qureshi A.T., Hayes D.J., Wu Q. (2013). Electrospun Bio-Nanocomposite Scaffolds for Bone Tissue Engineering by Cellulose Nanocrystals Reinforcing Maleic Anhydride Grafted PLA. ACS Appl. Mater. Interfaces.

[79] Rahatuzzaman M., Mahmud M., Rahman S., Hoque M.E. (2024). Design, Fabrication, and Characterization of 3D-Printed ABS and PLA Potentially for Tissue Engineering. Results Eng..

[80] Nemethova M., Auinger S., Small J.V. (2008). Building the Actin Cytoskeleton: Filopodia Contribute to the Construction of Contractile Bundles in the Lamella. J. Cell Biol..

[81] You R., Li X., Luo Z., Qu J., Li M. (2015). Directional Cell Elongation through Filopodia-Steered Lamellipodial Extension on Patterned Silk Fibroin Films. Biointerphases.

